# Rate-Dependent Painful Left Bundle Branch Block (PLBBB) in a Structurally Normal Heart

**DOI:** 10.7759/cureus.105638

**Published:** 2026-03-22

**Authors:** Daniela Barbosa, Inês Gomes Campos, Nuno Magalhães, Joel Ponte Monteiro

**Affiliations:** 1 Internal Medicine, Tâmega e Sousa Local Health Unit, Penafiel, PRT; 2 Cardiology, Tâmega e Sousa Local Health Unit, Penafiel, PRT

**Keywords:** acute myocardial infarction, beta-blockers, chest pain, heart rate, painful left bundle branch block

## Abstract

Painful left bundle branch block (PLBBB) is a rare and often underdiagnosed cause of exertional chest pain in patients without structural heart disease or coronary obstruction. We report the case of a woman in her fifties with well-controlled hypertension and no family history of sudden cardiac death, who presented with exertional chest tightness. During an exercise stress test, her typical symptoms appeared concurrently with a transient, rate-dependent LBBB, which resolved during recovery with normalization of the QRS. Cardiac biomarkers, echocardiography, and coronary angiography were normal, confirming a structurally normal heart. Beta-blocker therapy led to marked symptomatic improvement and good tolerance to a structured exercise program. Rate-dependent PLBBB is a benign but clinically relevant conduction disturbance that can mimic myocardial ischemia. Recognition of this entity is essential to avoid unnecessary invasive investigations and to guide appropriate management focused on heart rate control and symptom relief.

## Introduction

Left bundle branch block (LBBB) is an intraventricular conduction disorder characterized by delayed or disrupted electrical activation of the left ventricle, resulting from impaired conduction within the left bundle branch of the His-Purkinje system [1]. Its prevalence increases with age, rising from <1% at 50 years to approximately 6% by 80 years [2]. According to American College of Cardiology/American Heart Association/Heart Rhythm Society (ACC/AHA/HRS) guidelines, LBBB is defined electrocardiographically by a QRS duration ≥120 ms, broad or notched R waves in leads I, aVL, V5, and V6, absence of Q waves in I, V5, and V6, an R-wave peak time exceeding 60 ms in V5 and V6, and ST-T wave changes typically discordant with the QRS [3].

Clinically, LBBB gains particular significance when associated with chest pain, as it complicates the distinction between myocardial ischemia, coronary vasospasm, or structural heart disease. Transient LBBB with anginal symptoms, in the absence of obstructive coronary disease, defines painful left bundle branch block (PLBBB) syndrome- a rare condition of uncertain pathophysiology with no standardized management [4-6].

## Case presentation

We report the case of a woman in her fifties with a history of well-controlled hypertension, managed with an angiotensin II receptor antagonist, and a partial thyroidectomy. She had no family history of sudden cardiac death. She presented with intermittent, exertion-induced chest tightness, without prodromes or syncope, raising concern for possible cardiac ischemia. During an exercise stress test, she developed her characteristic symptoms, accompanied by progressive widening of the QRS complex with a LBBB morphology. During recovery, her symptoms resolved, and the QRS complex returned to normal. The episode was hemodynamically well tolerated, highlighting the transient nature of the conduction disturbance.

**Figure 1:**
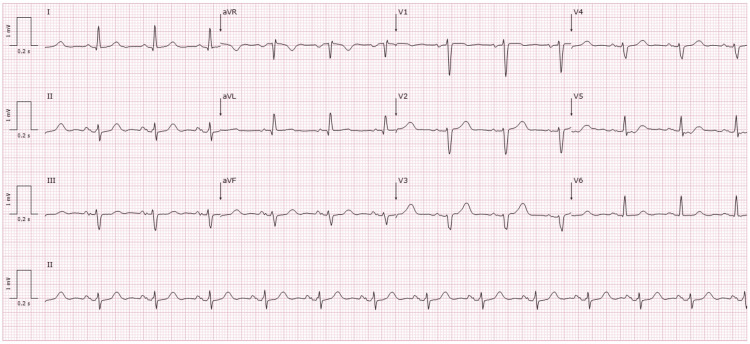
Baseline 12-lead electrocardiogram The electrocardiogram shows sinus rhythm, left axis deviation, and poor R-wave progression in the precordial leads.

**Figure 2:**
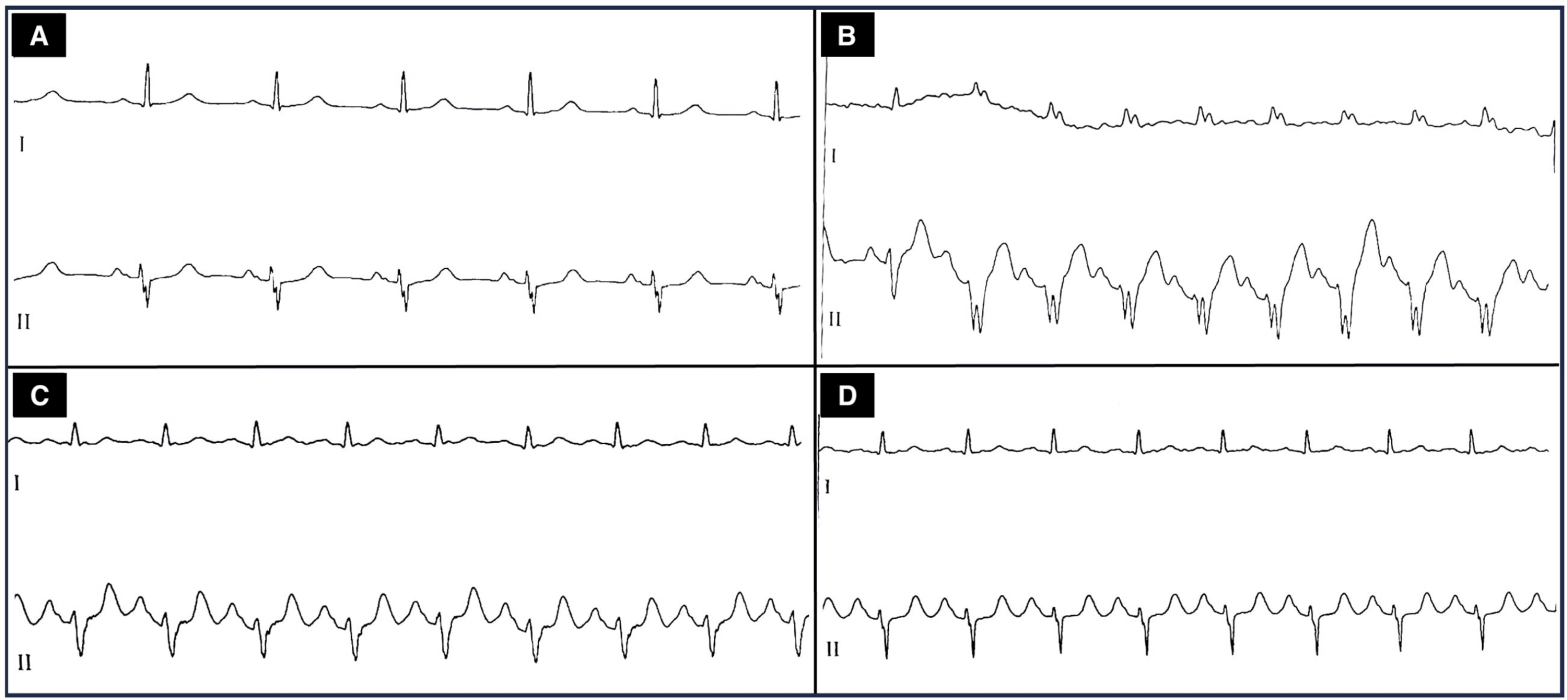
ECG tracing at the beginning of exercise showing LBBB (A); accentuation of the left bundle branch block (LBBB) with the Ashman phenomenon (B); and reduction of aberrant conduction with heart-rate slowing following exercise cessation (C, D).

Subsequent cardiology evaluation revealed no resting chest pain, stable vital signs, and a resting electrocardiogram (ECG) demonstrating sinus rhythm (Figure 1). A meticulous review of the exercise ECG tracings revealed that the arrhythmia corresponded to sinus tachycardia with Ashman phenomenon, manifesting as intermittent LBBB morphology (Figure 2). The patient became symptomatic at heart rates around 160 beats per minute (bpm), with symptoms progressively improving as the rate decreased and resolving upon normalization (heart rate around 100 bpm).

Bisoprolol 2.5 milligrams was initiated, resulting in a favorable symptomatic response. Follow-up myocardial perfusion scintigraphy revealed no evidence of ischemia, allowing for the safe introduction of a structured daily exercise program. This was well tolerated and led to further clinical improvement. After nine months of follow-up, the patient remained asymptomatic and had returned to a normal lifestyle.

## Discussion

The first case of LBBB associated with anginal chest pain and normal coronary angiography was reported by Vieweg et al. in 1976 [5], and subsequent reports have described similar phenomena, often characterized by abrupt onset and resolution of pain. LBBB has transitioned from a mere electrocardiographic curiosity to a clinically significant entity, increasingly acknowledged for its association with structural and functional cardiac abnormalities and the complex diagnostic and therapeutic challenges it poses in contemporary practice [3-7]. To standardize diagnosis, Shvilkin et al. [6] proposed specific criteria for PLBBB, including: abrupt onset of chest pain coinciding with the development of LBBB; resolution of symptoms with normalization of conduction; a normal 12-lead ECG before and after LBBB; absence of ischemia during stress testing; normal left ventricular function without other explanatory conditions; and a precordial S/T ratio < 1.8 with inferior axis correlation, all of which were present in the case under discussion [6,7]. 

Even in the absence of classical electrocardiographic patterns of acute myocardial infarction, the appearance of LBBB in association with chest pain should raise suspicion of myocardial ischemia. Given the worse prognosis associated with presumed new LBBB, the Sgarbossa criteria and their modifications are valuable tools for the accurate diagnosis of acute ischemia in atypical presentations [6]. The pathophysiology of PLBBB remains incompletely understood. Evidence suggests that LBBB, by disrupting ventricular synchrony, may reduce cardiac output and precipitate symptoms such as dyspnea and chest discomfort. Additionally, a heart rate threshold appears to modulate the onset of typical LBBB patterns, suggesting a chronotropic trigger [4,6]. 

No consensus exists regarding optimal management. Therapeutic strategies range from supervised exercise conditioning, beta-blockers, and calcium channel blockers to advanced interventions such as right ventricular pacing, biventricular resynchronization, and his-bundle pacing, the latter of which has shown promising efficacy in recent reports [4,6,8].

## Conclusions

PLBBB syndrome remains a rare and challenging condition, with mechanisms not yet fully understood. This case illustrates the dynamic relationship between exertion, rate-dependent conduction abnormalities, and symptom onset, with full resolution at rest. The patient’s hemodynamic stability and clear ECG correlation underscore the importance of careful evaluation. Early recognition is essential not only to guide management but also to distinguish PLBBB from other potentially serious conditions, such as myocardial ischemia, coronary vasospasm, or structural heart disease, which can present with similar symptoms. This case provides a valuable reference for understanding this uncommon clinical entity.
